# Structural Insights into Triglyceride Storage Mediated by Fat Storage-Inducing Transmembrane (FIT) Protein 2

**DOI:** 10.1371/journal.pone.0010796

**Published:** 2010-05-24

**Authors:** David A. Gross, Erik L. Snapp, David L. Silver

**Affiliations:** 1 Department of Biochemistry, Albert Einstein College of Medicine, Bronx, New York, United States of America; 2 Department of Anatomy and Structural Biology, Albert Einstein College of Medicine, Bronx, New York, United States of America; Universität Heidelberg, Germany

## Abstract

Fat storage-Inducing Transmembrane proteins 1 & 2 (FIT1/FITM1 and FIT2/FITM2) belong to a unique family of evolutionarily conserved proteins localized to the endoplasmic reticulum that are involved in triglyceride lipid droplet formation. FIT proteins have been shown to mediate the partitioning of cellular triglyceride into lipid droplets, but not triglyceride biosynthesis. FIT proteins do not share primary sequence homology with known proteins and no structural information is available to inform on the mechanism by which FIT proteins function. Here, we present the experimentally-solved topological models for FIT1 and FIT2 using N-glycosylation site mapping and indirect immunofluorescence techniques. These methods indicate that both proteins have six-transmembrane-domains with both N- and C-termini localized to the cytosol. Utilizing this model for structure-function analysis, we identified and characterized a gain-of-function mutant of FIT2 (FLL(157-9)AAA) in transmembrane domain 4 that markedly augmented the total number and mean size of lipid droplets. Using limited-trypsin proteolysis we determined that the FLL(157-9)AAA mutant has enhanced trypsin cleavage at K86 relative to wild-type FIT2, indicating a conformational change. Taken together, these studies indicate that FIT2 is a 6 transmembrane domain-containing protein whose conformation likely regulates its activity in mediating lipid droplet formation.

## Introduction

The ability to store triglycerides and other non-polar lipids in cytosolic lipid droplets is a ubiquitous biochemical attribute of eukaryotes. Lipid droplets consist of a core of neutral lipids surrounded by a phospholipid monolayer and an associated proteome, of which the perilipin family [1], triacylglyceride and diacylglyceride lipases [2], CGI-58 [3], and Fsp27 [4], [5] are some of the best characterized lipid droplet-associated proteins to date. Lipid droplets play an important role in the maintenance of energy homeostasis, serving as a dense energy reservoir during starvation conditions. Under conditions of excessive energy intake, triglyceride accumulation can lead to obesity and associated diseases such as type 2 diabetes, cardiovascular disease, and metabolic syndrome [6].

Lipid droplet catabolism is highly regulated. In adipocytes, lipid droplet triglyceride hydrolysis is mediated by the lipases desnutrin/ATGL/PNPLA2, hormone sensitive lipase and by other lipid droplet-associated proteins, such as the perilipin family and CGI-58 [1], [2], [3]. Recently, the involvement of autophagy has been implicated in lipid droplet catabolism in hepatocytes [7], adding to the complexity of mechanisms that play a role in regulating lipid droplet turnover. Conversely, little information is known about the process of lipid droplet formation. The physical process of forming lipid droplets is thought to occur at or within the endoplasmic reticulum (ER) membrane [8]. Several forward genetic screens have been conducted in model organisms or cells to identify proteins important in lipid droplet metabolism and have surprisingly revealed that more than 1% of genes in eukaryotic genomes are involved in lipid droplet biology [9], [10], [11]. These genetic screens have identified proteins that are involved in regulating lipid droplet lipolysis, neutral lipid biosynthesis, and various other factors which do not yet have assigned roles in lipid droplet biology. Many of these undefined factors may eventually be characterized as having roles in lipid droplet formation.

We have recently described the identification of an evolutionarily conserved family of ER-resident membrane proteins that we named Fat-Inducing Transcripts 1 & 2 which have since been re-named Fat storage-Inducing Transmembrane proteins 1 & 2 (FIT1/FITM1 and FIT2/FITM2) [12]. FIT2 is anciently conserved back to *S. cerevisiae*, while FIT1 is found only as far back as boney fish. FIT1 is primarily expressed in skeletal muscle, with lower levels found in heart, and FIT2 is ubiquitously expressed in tissues, with the highest levels in white and brown adipose tissue.

FIT proteins are not located on lipid droplets, but exclusively found in the ER, the presumed site of lipid droplet formation. As the name implies, when these proteins are expressed in cells or in mouse liver, they induce triglyceride droplet accumulation without inducing triglyceride biosynthesis—an enzymatic reaction carried out by the diacylglycerol O-acyltransferase (DGAT) family of ER-resident membrane proteins [13], [14], [15]. Additionally, FIT proteins do not play a known role in lipolysis [12]. Knockdown of FIT2 in 3T3-L1 adipocytes or in zebrafish resulted in a significant decrease in lipid droplet accumulation, indicating an important role of FIT2 in lipid droplet accumulation in these models.

FIT proteins are unique in their biochemical mode of action in that they mediate the partitioning of triglyceride from the ER into cytosolic lipid droplets [12]. However, the mechanism by which FIT proteins mediate this process is not known. An impediment to understanding this process is the fact that FIT proteins do not share homology to known proteins or protein domains, making it difficult to infer a mechanism based on primary sequence. In addition, no structural information is available for FIT proteins to date. To gain a better understanding of the mechanism by which FIT proteins operate, we present here experimentally determined topological models for this unique family of proteins followed by structure-function analysis of the anciently conserved FIT2 protein revealing a specific group of conserved amino acids that play an important role in regulating FIT2 conformation and function.

## Results

### FIT1 and FIT2 are six-transmembrane-domain-containing proteins

FIT1 and FIT2 comprise a unique family of proteins that do not have homology to known proteins or protein domains found in any species. In order to create a foundation for structure-function analysis of FIT proteins, we sought to create a topological model of FIT proteins by first examining FIT2 as the archetype of the FIT family of proteins since it is the more ancient and conserved form and is expressed in all mammalian tissues, with the highest levels in white and brown adipose tissue [12]. The topology of FIT2 was previously predicted by the TMHMM algorithm to contain either five or six transmembrane domains [12].

**Figure 1:**
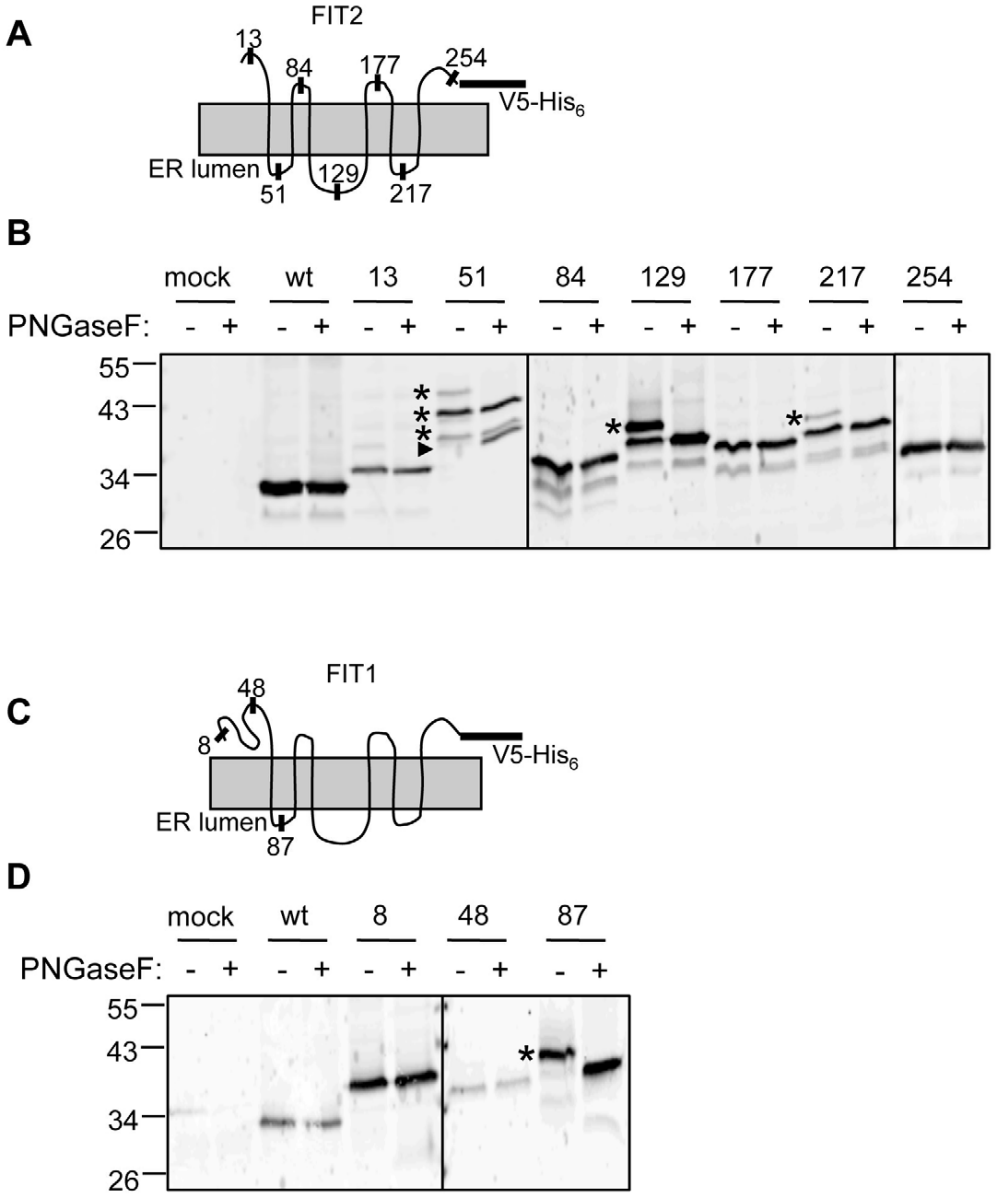
Membrane topology of murine FIT2 and FIT1. **A and C**, FIT2 and FIT1 topological models with N- and C-termini oriented toward the cytosol and six transmembrane domains with a large second luminal loop. The amino acid positions of insertion of FNF tags for the determination of topology by glycosylation site mapping and indirect immunofluorescence are indicated. As indicated, all constructs contain C-terminal V5 and polyhistidine epitope tags. **B and D**, Increased molecular weights of specific FNF-V5 constructs relative to wild-type FIT2 or FIT1 (indicated by asterisks) detected by Western blot analysis indicated potential glycosylation and luminal orientation. Decreased molecular weight of constructs following PGNaseF treatment indicated glycosylation and luminal orientation. Asterisks indicate glycosylated forms of FIT2 and FIT1 constructs. The multiple asterisks indicated for the FIT2 construct 51 is due to two tandem FNF glycosylation sites inserted into that construct (arrow indicates non-glycosylated construct 51). B and D are representative of two independent experiments.

In order to determine the orientation of the N- and C-terminus and the number and orientation of transmembrane domains, we performed epitope tag-glycosylation site mapping, a method that was successfully used to determine the topology of the ER-resident multispanning membrane protein Insig-1 [16]. In this method, a glycosylation consensus motif can be engineered into predicted cytosolic and luminal portions of ER-resident membrane proteins. Only inserted motifs that are exposed to the ER lumen can become accessible to glycosyltransferases in the ER; therefore, glycosylation indicates a luminal orientation. A glycosylation consensus sequence (NGT) flanked by a FLAG tag (DYKDDDDK) on each side (FLAG-NGT-FLAG, abbreviated FNF) was inserted into each of the possible luminal and cytosolic loops of C-terminally V5- and polyhistidine-tagged FIT2 (designated FIT2-V5, Fig. 1A). Each of these constructs was expressed in HEK293 cells and all were functional in terms of the ability to form lipid droplets (Fig. S1). Western blot analysis showed that FNF tags inserted at amino acid positions 51, 129 and 217 resulted in molecular weight shifts greater than the 19 amino acid addition of the FNF tag, likely indicating glycosylated FIT2-V5 (Fig. 1B). Insertions at amino acids 13, 84, 177, and 254 did not result in molecular weight shifts corresponding to greater than 19 amino acids–the molecular weight of the FNF tag (Fig. 1B). To prove that these molecular weight shifts were the consequence of glycosylation, cell lysates were treated with the endoglycosidase PNGaseF in order to cleave *N*-linked glycans. Lysates from cells expressing FIT2 constructs with FNF insertions at positions 51, 129, and 217 that were treated with PNGase F decreased in molecular weight, consistent with glycosylation and the localization of these domains to the ER lumen. PNGaseF treatment did not affect the apparent molecular weight of FNF insertions at 13, 84, 177, or 254, indicating that these loops are cytosolic (Fig. 1B).

Murine FIT1 and FIT2 are 35% identical and 50% similar [12]; however, FIT1 (292 amino acids) has 30 extra amino acids at its N-terminus and has limited homology with FIT2 (262 amino acids) in transmembrane domain 1. Furthermore, Kyte-Doolittle plots and TMHMM prediction models conflicted in the definition of transmembrane domain regions [12]. The reason for this is that the extra 30 amino acids at the N-terminus comprises a hydrophobic tract of amino acids that could be a seventh transmembrane domain or exist as a cytosolic domain. Therefore, we sought to confirm the topological model for the most poorly aligned part of FIT1. Using a similar approach for determining FIT2 topology, FNF tags were inserted at amino acid positions 8, 48, and 87 of FIT1 (Fig. 1C). Each construct was transiently expressed in HEK293 cells and tested for the presence of glycosylation as described above. When an FNF insertion was made at amino acid 8, FIT1-V5 mobility is reduced on SDS-PAGE, suggesting glycosylation (Fig. 1D). However, PNGaseF treatment clearly indicated that FNF at position 8 was not glycosylated, but that FNF at position 87 was glycosylated (Fig. 1D). The molecular weight shift observed for the FNF construct at position 8 suggests a structured N-terminus of FIT1 that is altered by this particular FNF insertion (Fig. 1D). These experiments showed that the FIT1 N-terminus and domains containing FNF insertions at amino acids 8 and 48 were not glycosylated, and thus likely exposed to the cytosol, while the domain containing a FNF tag at amino acid 87 was glycosylated, indicating that it is located in the ER lumen (Fig. 1D).

**Figure 2:**
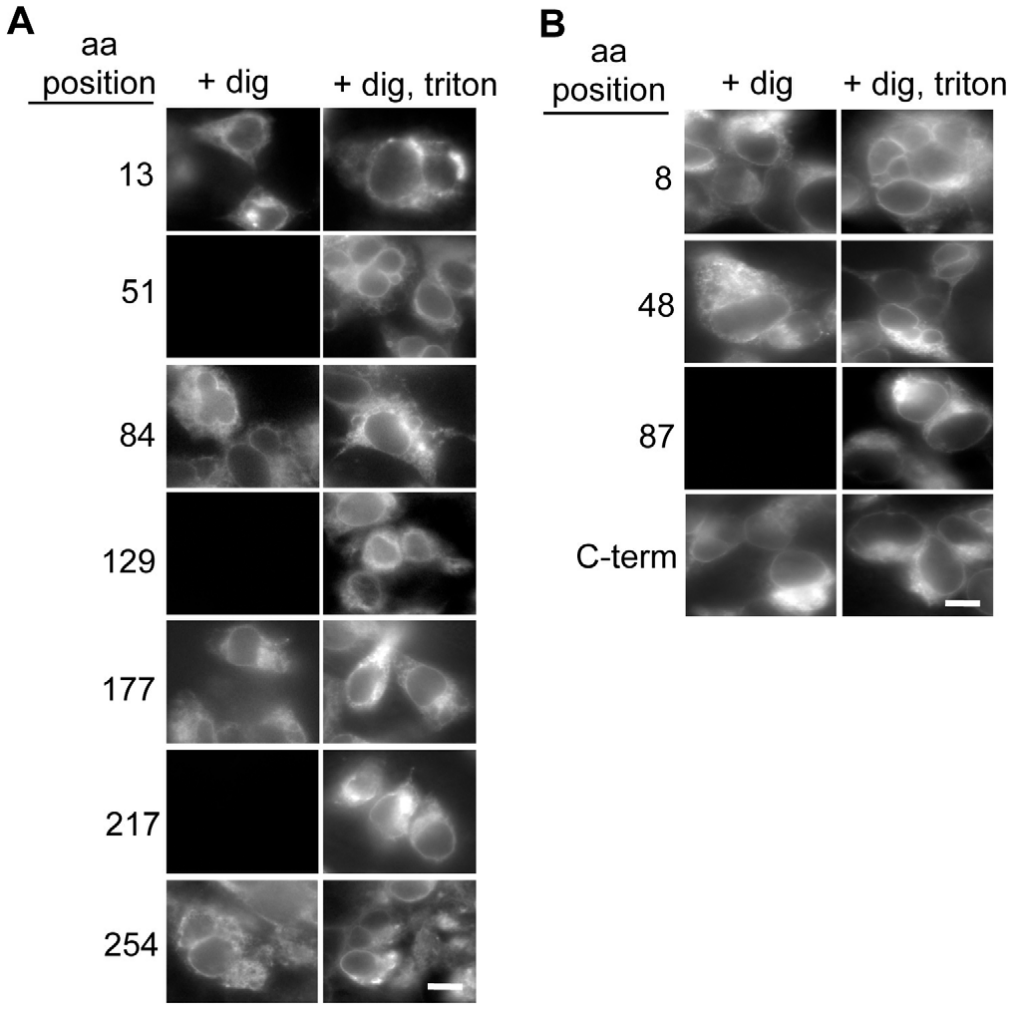
**A**, Indirect immunofluorescence microscopy of FIT2-V5 FNF insertions. Cells expressing various FIT2-V5 FNF mutants were fixed, permeabilized with digitonin with or without Triton X-100, and were incubated with FLAG antibody to detect the FLAG epitope. Cytosolic-oriented loops are detected in digitonin-permeabilized cells. Luminal-oriented loops are detected in cells permeablized with Triton X-100. **B**, Indirect immunofluorescence microscopy of FIT1-V5 FNF insertions. Cells were treated as in **A**, except that cells expressing FIT1-V5 without an FNF insertion were incubated with a V5 antibody to determine the orientation of the C-terminus. Images are representative of three independent experiments (scale bar: 5 µm).

To confirm the orientation of each transmembrane domain of FIT2 and FIT1 by an independent method, we used selective permeabilization of the plasma membrane in conjunction with indirect immunofluorescence microscopy to the FLAG epitope in each FNF construct. At low concentrations of digitonin (20 µM), only the plasma membrane, and not the ER can be permeabilized [17], while in the presence of 0.1% Triton X-100, both plasma membrane and ER membranes are permeabilized. We confirmed in our assays that we were achieving plasma membrane-selective permeabilization using the endogenously expressed ER-resident membrane protein calnexin as a control (Fig. S2). HEK293 cells were transiently transfected with each FIT2-V5 or FIT1-V5 FNF construct described above and were processed for indirect immunofluorescence using digitonin treatment with or without Triton X-100. Indirect immunofluorescence microscopy studies clearly confirmed the glycosylation studies (Fig. 2A, B) and indicated that the six-transmembrane domain topological models of both FIT1 and FIT2 were correct, in which both proteins have their N- and C-termini facing the cytosol (Fig. 1A, C).

### Identification of a FIT2 Gain-of-Function Mutant

**Figure 3:**
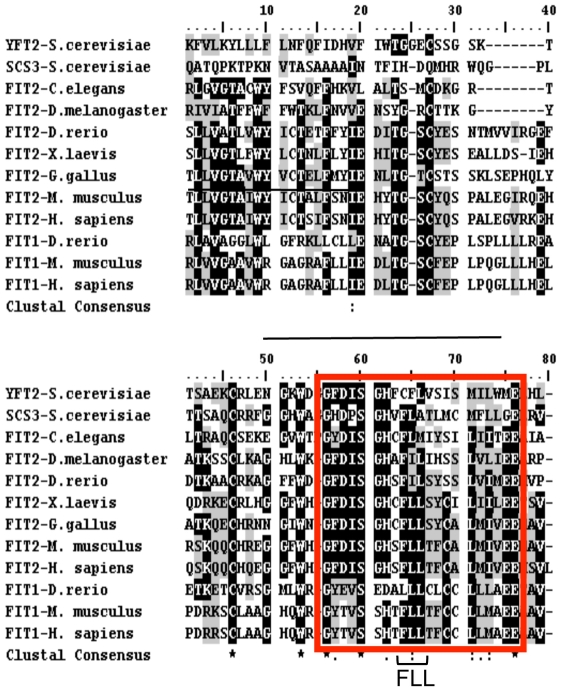
A conserved tract of amino acids in transmembrane domain 4 of FITs. Alignment of partial sequences of FIT2 and FIT1 from various species showing the “FIT signature sequence” is indicated within the red box. The most conserved group of residues in this domain comprises the FLL sequence, whose hydrophobic character was retained throughout evolution. Based on our experimentally determined topological model of FIT proteins (Figure 1 and 2), the FIT signature sequence is located in transmembrane domain 4.

**Figure 4:**
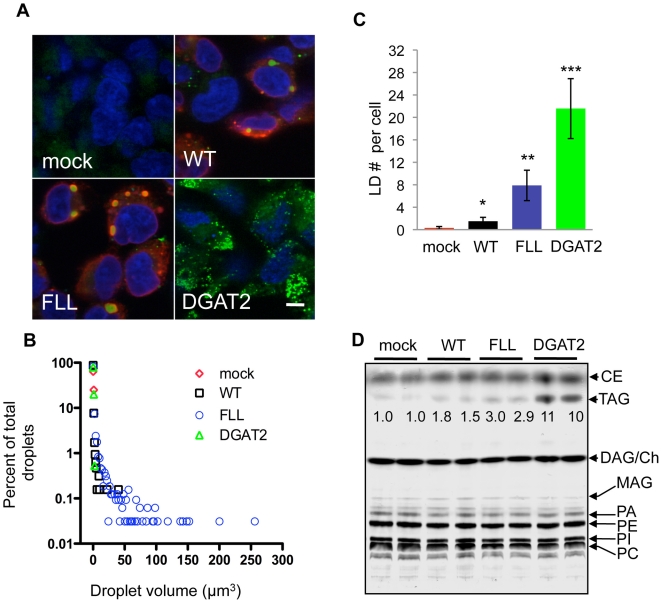
FLL(157-9)AAA FIT2-V5 is a gain-of-function mutant. **A**, Confocal fluorescence microscopy of lipid droplets in HEK293 cells transiently transfected with wild-type FIT2-V5 (WT), FLL(157-9)AAA FIT2-V5 (designated FLL), human DGAT2, or mock transfected control. Images are representative of three independent experiments. Cells were stained for lipid droplets (green) and FIT2-V5 (red), and nuclei (blue). **B**, Histogram of lipid droplet volumes. HEK293 cells were set up as in **A** and subjected to confocal fluorescence microscopy. Z-stacks were captured and 3D renderings were constructed. Data was analyzed using Velocity software (Perkin Elmer). Analysis was performed on 10 independent 3D renderings from three independent experiments. **C**, Quantification of lipid droplets per cell is shown (mock vs WT*, FLL**, and DGAT2***: p<3×10^−4^, 2.5×10^−5^, 4.9×10^−7^, respectively). The data are presented as mean ± s.d. **D**, Quantification of cellular triglyceride concentration in cells expressing the indicated constructs. DGAT2 or mock transfected were used as positive and negative controls, respectively. The data are presented as fold-increase over mock control and are representative of three independent transfections.

**Figure 5:**
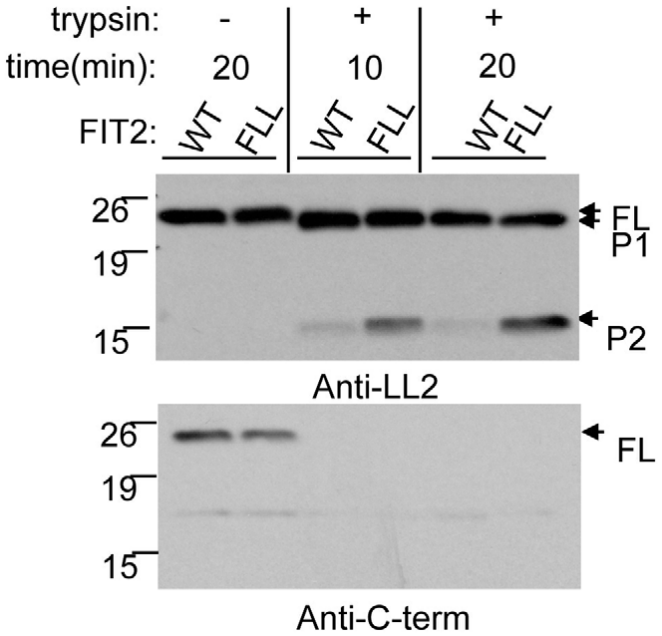
FLL(157-9)AAA FIT2 has an altered conformation compared to wild-type FIT2. FLL(157-9)AAA FIT2 (FLL) and FIT2 (WT) were transfected into cells and limited trypsin digestion time-courses were performed on both of these constructs. Results were analyzed by Western blot using antibodies directed to the luminal loop 2 of FIT2 (designated anti-LL2) and the C-terminus (designated anti-C-term). FLL was expressed to similar levels as WT (lanes 1 and 2). Two proteolytic products were detected and designated P1 and P2. The amount of P2 is increased for FLL following trypsin digestion. As a control for the efficiency of digestion of each construct, blots were reprobed with the anti-C-term antibody (lower panel) that detects the cytosolic-localized FIT2 C-terminus. FL designates full length FIT2. The Western blots shown are representative of three independent experiments.

Transmembrane domain 4 of FITs contains the most highly conserved residues throughout the FIT protein family which can be considered a FIT signature sequence (Fig. 3). Using FIT2-V5 as an archetype for our studies of FIT function, we performed alanine mutagenesis on this domain. Single or paired conserved residues were mutated to alanine, expressed in HEK293 cells, and lipid droplets were visualized by fluorescence confocal microscopy in order to characterize alterations in phenotype. To visualize lipid droplets we utilized the neutral lipid fluorescent dye BODIPY 493/503, commonly used to visualize and quantify lipid droplets due to its high specificity for lipid droplets [18]. This stain was recently and successfully used in a high-throughput forward genetic screen to identify genes important in lipid droplet biology [10]. In order to determine that cells having lipid droplets expressed FIT2, FIT2 expressing cells were identified by immunofluorescence. Of the mutants generated, FLL(157-9)AAA (abbreviated as FLL) showed a pattern of increased number of lipid droplets and larger lipid droplets compared to wild-type FIT2 when subjected to BODIPY 493/503 staining and confocal fluorescence microscopy (Fig. 4A). The observed lipid droplet phenotype of cells expressing FLL could not be explained by either expression level of FLL relative to wild-type FIT2, its subcellular localization (Fig. 5 lanes 1 & 2, Fig. S3, and Fig. S5), or transfection efficiency (90% for both wild-type and FLL). Mock transfected cells served as a negative control for lipid droplet staining, since HEK293 cells rarely exhibited lipid droplets. DGAT2 overexpression served as a positive control and resulted in an abundance of small lipid droplets. Quantification of lipid droplet number and size clearly indicated that expression of the FLL mutant resulted in a 5-fold increase in mean lipid droplet size (WT, 0.8±0.2 µm^3^; FLL, 4.0±0.3 µm^3^; p = 3.68×10^−6^) (Fig. 4B) and a 5-fold increase in lipid droplet number compared to cells expressing wild-type FIT2 (Fig. 4C). As expected, mock-transfected cells rarely exhibited droplets, while DGAT2-expressing cells had an average of 21 small lipid droplets per cell (average volume, 0.55±0.08 µm^3^) (Fig. 4B, C). Despite the 5-fold increase in both lipid droplet number and size in cells expressing FLL mutant, triglyceride levels were only increased 1.8-fold compared to cells expressing WT FIT2. Triglyceride levels in cells expressing FLL or WT FIT2 were moderately increased relative to mock-transfected control cells (3-fold and 1.6-fold, respectively) compared to the greater than 10-fold increase in cells expressing DGAT2 (Fig. 4D). This finding is consistent with our previous finding that FIT2 does not mediate triglyceride biosynthesis, a biochemical function mediated by the DGAT enzymes [13], but rather mediates the partitioning of cellular triglycerides into lipid droplets [12]. No significant changes in various phospholipid species were found (Fig. 4D). Taken together, the data indicate that the FLL mutation imparts a gain-of-function on FIT2 resulting in increased lipid droplet accumulation.

### FLL(157-9)AAA FIT2 has a conformational change

We next sought to explain the gain-of-function phenotype of the FLL mutant. We hypothesized that the FLL mutant has an activating conformational change relative to wild-type FIT2. We tested this hypothesis using a limited trypsin digestion method previously used to determine conformational changes in ER-resident polytopic membrane proteins in response to lipids [19], [20]. The readout for this technique is to simply identify any alteration in trypsin resistant fragments by Western blot analysis using an antibody to a luminal domain encompassing a trypsin resistant epitope. Therefore, changes in tryptic digestion pattern are a surrogate marker for changes in protein conformation.

Taking advantage of our experimentally determined topological model of FIT2, we generated an antibody (anti-FIT2 LL2) to the second luminal loop of FIT2 (Fig. S4). Wild-type and FLL mutant FIT2 were expressed in HEK293 cells followed by digitonin permeabilization of the plasma membrane and digested with trypsin for various times. Limited trypsin digestion of FIT2 resulted in the appearance of two fragments designated P1 and P2 (Fig. 5). It is important to note that full length FIT2 is reduced in its entirety to P1 at the 10 min time point of digestion, but a quantitatively lower amount of P2 is generated compared to P1, indicating that generation of P1 precedes the generation of P2. Importantly, the amount of the 15 kD P2 fragment of the FLL mutant is increased compared to the P2 fragment generated from wild-type FIT2, indicating a conformational change in FLL relative to wild-type FIT2. When blots were re-probed with an antibody to the C-terminal 13 amino acids of FIT2, an epitope that is localized to the cytosol, both FLL mutant and wild-type were digested to similar levels in the presence of trypsin (Fig. 5, bottom panel, lanes 3–6). Importantly, FLL mutant and wild-type FIT2 were expressed to similar levels (Fig. 5, lanes 1 and 2).

**Figure 6:**
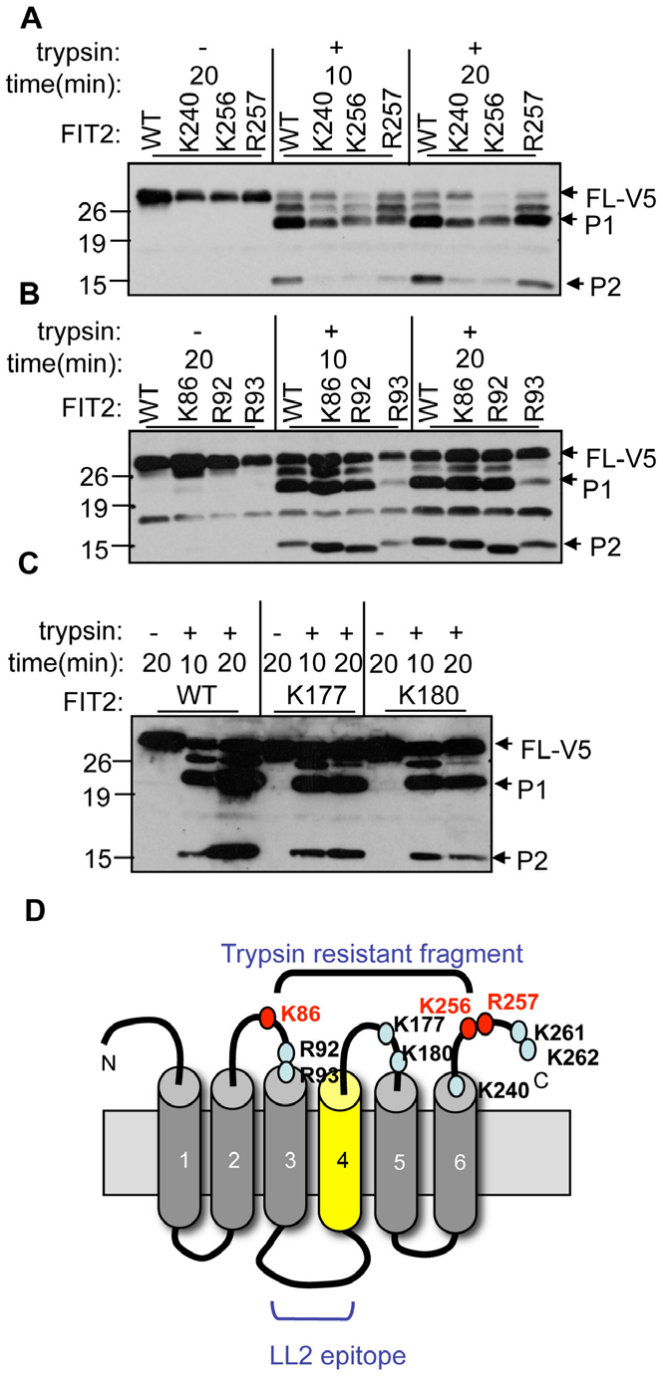
Mutational analysis to define the P1 and P2 fragment trypsin cleavage sites. **A**, Cells were set up as in Fig. 5 by expressing either K240A, K256A, R257A (all constructs are V5 tagged) and compared to wild-type FIT2-V5 (WT) in limited trypsin digestion time course assays as indicated. Additional bands were detected with the anti-LL2 antibody due to the presence of lysine and arginine residues in the C-terminal V5 tag. Note the molecular weight shift in the P1 fragment of K256A and R257A, but not of K240A, indicating that K256 or R257 are likely cleavage sites giving rise to P1. Also of note is the lower amount of P2 generated in these mutants compared to wild-type correlating with lower expression levels of these constructs. **B**, Cells were set up as in **A** to examine the tryptic pattern of K86A, R92A and R93A compared to wild-type. Of note are the decreased molecular weights of the P2 fragments of K86A and R92A, but not of R93A compared to WT, indicating that cleavage likely occurs at K86 (and K256/R257) to generate the P2 fragment. **C**, Cells were set up as in **A and B**. K177A or K180A did not affect the size of P2, indicating that these residues are not cleaved by trypsin in WT FIT2. **D**, Topological model showing the possible cytosolic trypsin-susceptible residues of FIT2 in the limited trypsin digestion assay. Based on the above data, residues in red indicate those that are cleaved to form proteolytic products P1 and P2. Residues in blue are those that are not cleaved by trypsin in wild-type FIT2. These data are representative of two independent experiments.

We next wanted to determine the cleavage sites of FIT2 that gave rise to the P1 and P2 fragments. Since trypsin cleaves C-terminal to lysine and arginine, we generated alanine mutations in cytosolic lysines and arginines in FIT2-V5 and screened for changes in P1 and P2 fragment sizes. Mutagenesis of K240 did not alter the molecular weight of the P1 fragment, despite being expressed to lower levels than wild-type FIT2. Mutation of K256 or R257 resulted in an increased molecular weight of P1 fragment compared to wild-type FIT2, indicating that both K256 and R257 are cleaved by trypsin and likely constitute the C-terminus of the P1 and P2 fragment (Fig. 6A). Mutation of K86 resulted in a P2 fragment having a smaller molecular weight compared to wild-type FIT2 (Fig. 6B). Mutation of R92 resulted in a further, minor reduction in molecular weight of P2 compared to wild-type, that is likely consistent with cleavage at R93. Mutation of R93 did not affect the molecular weight of the P2 fragment and resulted in a P2 fragment of similar molecular weight as found in wild-type, indicating that R93 is not a cleavage site in wild-type FIT2 and that R93 is likely utilized only in the R92A mutant, but not in wild-type FIT2 (Fig. 6B). Cleavage at K177 or K180 is unlikely because this would result in a P2 fragment having a molecular weight of approximately 9 kD. Indeed, mutation of these residues did not result in a change in molecular weight of the P2 fragment (Fig. 6C). Taken together, these data indicate that the N- and C-terminus of the P2 fragment is likely generated by cleavage at K86 and K256/R257, respectively (Fig. 6D), and since the generation of P1 precedes the generation of P2, the conformational change occurring in the FLL mutant likely occurs around residue K86 leading to increased trypsin susceptibility at K86. Since these studies to identify the trypsin cleavage sites utilized V5-tagged FIT2 constructs, as a control we wanted to test whether the V5 tag could influence the tryptic pattern seen in the FLL mutant. Importantly, the FLL-V5 tagged construct also showed an increased amount of P2 fragment compared to FIT2-V5 wild-type (Fig. S5). In summary, these results indicate that the FLL mutant has a conformational change relative to wild-type FIT2 that resulted in a gain-of-function phenotype.

## Discussion

FIT1 and FIT2 constitute a novel family of evolutionarily conserved proteins that do not share sequence similarity to known proteins or domains and have a unique function [12]. FIT proteins are exclusively located in the endoplasmic reticulum membrane, the presumed site of lipid droplet formation, and are not found on lipid droplets. In a previous study, we showed that FIT proteins mediate the partitioning of triglyceride into cytosolic lipid droplets without mediating triglyceride biosynthesis. However, the mechanism by which FIT proteins partition triglyceride into lipid droplets is not known. Our goal in the present study was to gain an understanding of this mechanism by experimentally solving the topological model of FIT proteins and determining the functional relevance of evolutionarily conserved residues. We found, by using two separate methods, glycosylation site insertion, and indirect immunofluorescence, that both FIT1 and FIT2 have six transmembrane domains with both N- and C-termini located in the cytosol. This topological model of FIT1 and FIT2 bears resemblance to the topology of the Insig family of ER-resident membrane proteins involved in regulating sterol homeostasis [16].

Comparative sequence analysis revealed a highly conserved tract of residues located in transmembrane domain 4 that we designated the “FIT signature sequence.” In order to understand the functional role of the FIT signature sequence we made alanine substitutions of individual or combinations of residues of the signature sequence in FIT2. Our rationale for focusing on FIT2 rather than FIT1 was that FIT2 is the anciently conserved homolog of the FIT family and is ubiquitously expressed in mammalian cells and tissues. Alanine replacements of residues comprising L164, MIV165–167, E168, or E169 resulted in loss of function and protein instability (D. Gross and D. Silver, unpublished observations). Without structural information of FIT2, these mutations are not functionally informative. However, alanine substitution of FLL157–159 (designated FLL) resulted in a gain-of-function when expressed in cells, making this mutant informative in ascertaining FIT2 function. Cells expressing this mutant version of FIT2 exhibited a 5-fold increase in both lipid droplet number and size, but only a 1.8-fold increase in cellular triglyceride relative to wild-type FIT2. The finding that cells expressing the FLL mutant relative to wild-type FIT2 had a disproportionate increase in lipid droplet numbers and size compared to total cellular triglyceride levels is not surprising and is explained by the observation that FIT2 does not mediate triglyceride biosynthesis but rather the re-distribution or partitioning of cellular triglycerides into lipid droplets [12]. The modest increase in cellular triglycerides in cells expressing FIT2 is similar to what has been described in cells expressing the lipid droplet protein perilipin, which dramatically enhances lipid droplet accumulation in cells disproportionately to total cellular triglyceride accumulation without augmentation of triglyceride biosynthesis [21]. It is likely that the half-life of triglyceride in lipid droplets is longer than in cellular membranes, giving rise to modest increases in cellular triglycerides in response to FIT overexpression.

**Figure 7:**
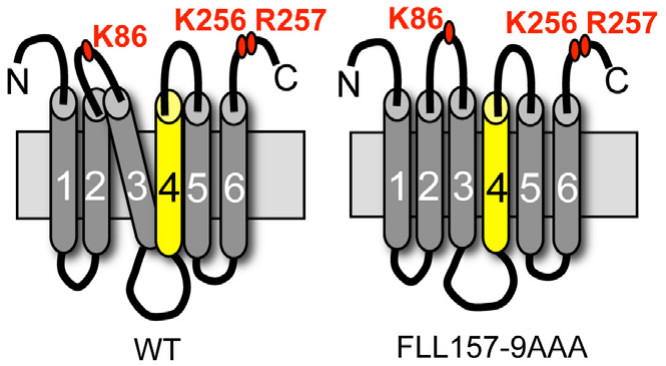
Model for conformational change in FIT2 FLL mutant. The FIT2 FLL mutant has an altered conformation relative to wild-type FIT2 that results in increased solvent accessibility of residues in cytosolic loop 2 containing residue K86. This conformational change may facilitate increased partitioning of triglyceride into nascent cytosolic lipid droplets or allow for the creation of larger lipid droplets by undetermined mechanisms.

We hypothesized that the FLL mutant has an altered conformation relative to wild-type FIT2. We used limited trypsin digestion to test this hypothesis. To perform this method, which has been used successfully to study lipid-induced changes in two other ER resident membrane proteins, HMG-CoA Reductase and SCAP [19], [20], we needed to develop an antibody to a luminal loop near the FLL sequence. Taking advantage of our topological model of FIT2, we developed antibodies to the second luminal loop of FIT2. Our studies revealed that trypsin digestion of FIT2 and the FLL mutant resulted in a trypsin resistant fragment of ∼15 kDa (designated P2) due to cleavage at K86 and K256/R257. Importantly, the amount of P2 fragment generated by digestion of the FLL mutant was increased relative to wild-type FIT2, indicating an altered conformation of this mutant. We interpret these findings to indicate that the FLL residues in transmembrane domain 4 stabilize a specific conformation that attenuates or regulates FIT2 activity. Substitution of these large non-polar side chains with methyl groups either increases solvent accessibility around K86 and/or permits greater antibody affinity in the second luminal loop of FIT2, as indicated in limited trypsin digestion experiments, correlating with increased FIT2 activity (Fig. 7). It is tempting to speculate that the activity of wild-type FIT2 is regulated by conformational changes induced by ER membrane lipids or interaction with other ER proteins. Such modes of regulation have been clearly demonstrated for Insig, SCAP and HMG-CoA Reductase [22], [23]. Whether FIT2 is similarly regulated will require further study into this recently identified family of proteins.

## Materials and Methods

### Plasmid constructs

V5/His_6_-tagged murine FIT1 or FIT2 in the pcDNA3.1-V5/His_6_ vector (Invitrogen Corp) were used as a template for all mutagenesis reactions. Insertion of FLAG tags flanking NGT glycosylation consensus sequences (FLAG-NGT-FLAG, FNF tags) or generation of FLL157–159AAA was performed by designing primers for overlap-extension PCR. All desired mutations were verified by sequence analysis.

### Generation of anti-LL2 FIT2 antibody

Polyclonal antibodies were raised in rabbit against 15 amino acids from the second luminal loop of FIT2 (ALEGIRQEHRSKQQC) and validated for specificity by using lysates from HEK293 cells expressing FIT2 (Fig. S4).

### Cell culture and transfection of cell

HEK293 cells from the American Type Tissue Culture Collection were culture in Dulbecco's modified Eagle's medium (DMEM) with 10% Fetal Bovine serum (FBS) and 1X penicillin/streptomycin (DMEM-FBS) at 37°C with 5% CO_2_. Transient transfections were performed using Lipofectamine 2000 according to manufacturer's instructions (Invitrogen Corp.).

### PNGasF treatment

HEK293 cells were plated, and transiently transfected with indicated wild-type or FNF FIT1-V5 or FIT2-V5 constructs on day 0. 18 hrs post-transfection, cell lysates were prepared and 10 µg of protein from each lysate was digested with or without 10,000 units of PNGaseF (New England Biolabs) for 1 hr at 37°C according to Zelenski et al. [24]. Samples were not boiled prior to PNGaseF treatment. Reactions were stopped by addition of Laemmli buffer and analyzed by immunoblot analysis using anti-V5 antibody.

### Immunoblot analysis

Samples were separated by SDS-PAGE on 10, 12, or 15% polyacrylamide gels, transferred to nitrocellulose membranes (Bio-Rad) and incubated with indicated antibodies.

### Indirect immunofluorescence microscopy

HEK293 cells were plated on 25-mm glass bottom dishes (Willco), transfected and allowed to express protein for 18 hours or form lipid droplets for 36 hours. Cells were then washed twice with PBS, stained with 12 µg/ml BODIPY 493/503 (Invitrogen) in DMEM-FBS for 10 minutes at room temperature and 2.5 µg/ml Hoechst 33342 (Invitrogen) in DMEM-FBS for 25 minutes at room temperature where indicated. Cells were then washed twice more with PBS and fixed for 15 minutes at room temperature in 3.7% methanol-free formaldehyde in PBS. Cells were washed twice again with PBS and permeabilized sequentially in PBS with 20 µM digitonin for 1.5 minutes with or without 0.1% Triton X-100 for 3 minutes at room temperature. Cells were washed three times with PBS between and after permeabilization procedures. Following selective permeabilization, cells were blocked with 5% filtered goat serum in PBS for 10 minutes at room temperature. Post-blocking, cells were incubated for 1 hour at room-temperature in PBS with 1∶400 dilution of rabbit anti-calnexin C-terminus (Sigma-Aldrich), rabbit anti-calnexin N-terminus (Stressgen), mouse anti-FLAG (Sigma-Aldrich), rabbit anti-LL2 FIT2, and/or mouse anti-V5 (Invitrogen Corp) antibodies. Cells were washed three times in PBS and incubated with 1∶500 dilution of goat anti-rabbit or donkey anti-mouse AlexaFluor488, AlexaFluor562 or AlexaFluor680. Cells were washed three more times, resuspended in PBS and analyzed on an Olympus IX81 electronically motorized fluorescence microscope with a 60X N.A. 1.4 Phase 3 objective.

### Measurement of lipid droplet number and size

HEK293 cells were plated on 25-mm glass bottom dishes, transfected (mock, FIT2-V5, FLL, or DGAT2), and allowed to form lipid droplets for 36 hours. Cells were fixed and processed for indirect immunofluorescence detection of FIT2-V5, then treated with 12 µg/ml BODIPY 493/503 (Invitrogen Corp) in DMEM-FBS for 10 minutes at room temperature and 2.5 µg/ml Hoechst 33342 (Invitrogen Corp) in DMEM-FBS for 25 minutes at room temperature. Cells were then washed twice in PBS. A Zeiss Live/DuoScan confocal microscopy using a 63X NA = 1.4 OIL objective and AIM 4.2 software was used to generate z-stacks and reconstruct three-dimensional projection images of cells. 10 random fields were examined and 100 optical z-sections were generated for each field. Both lipid droplet volumes and lipid droplet number per cell expressing FIT2 (based on detection of V5) were quantified using Velocity software (Perkin Elmer). The threshold for identifying lipid droplets was established as intensities greater than 20% of maximum intensity. This cutoff allowed the program to eliminate non-specific, diffuse staining that is localized to the endoplasmic reticulum and was considered background.

### Quantification of total cellular triglycerides

HEK293 cells were plated in 10-cm dishes, transfected, and allowed to form lipid droplets for 36 hours. Cells were then washed twice with PBS and extracted twice on the plate with 3∶2 hexane/isopropanol (HIP). HIP solutions were transferred to glass-bottom tubes, dried down under a stream of nitrogen and re-dissolved in solvent #1 and spotted onto glass silica TLC plates (EMD Biosciences) pre-washed with 60∶35∶8 chloroform/methanol/water. Lipid standards for various lipid species were also run on TLC plates. The plate was run to one-third of the plate's height in solvent #1 (25∶15∶4∶2) chloroform/methanol/acetic acid/water, removed and dried completely. The plate was then run in solvent #2 (60∶40∶4 heptanes/isopropyl ether/acetic acid) until the solvent reached the top of the plate. The plate was again allowed to dry completely and then run to one-third height in solvent #3 (45∶15∶10 ethyl acetate/isooctane/acetic acid). The plate was dried, sprayed to saturation with 3% cupric-acetate/8% phosphoric acid in water using a glass sprayer and allowed to dry overnight at room temperature. The TLC plate was developed by activating the cupric acetate at 80°C for 3 minutes and immediately transferred to 200°C in order to develop the plate until all samples were visible. Plates were scanned and band densities were analyzed by Quantity One 4.6.1 software image analysis software (BioRad).

### Limited trypsin proteolysis

This protocol was modified from Brown et al. [19]. Briefly, HEK293 cells were transiently transfected in tissue culture plates with indicated constructs on day 0 and grown in DMEM-FBS. 18 hrs post-transfection, cells were washed twice with PBS, transferred to 1.5 ml Eppendorf tubes by agitation and pipetting, pelleted and permeabilized for 1.5 minutes with 20 µM digitonin dissolved in PBS containing a protease inhibitor cocktail. Cells were washed twice more with PBS, resuspended in 500 µl PBS, and 0.8 µg trypsin in 4 µl PBS was added where indicated and incubated for various times at 31°C. Reactions were quenched with 40 µg Soybean Trypsin Inhibitor (Sigma-Aldrich) in 10 µl PBS, membranes were pelleted at 16,000×*g* for 1 minute at room temperature and lysed at 4°C for 1 hour in 500 µl RIPA buffer. Cell debris and nuclei were pelleted by centrifugation at 5,000×*g* for 10 minutes at 4°C and the supernatant protein concentration was determined by Bradford assay. For immunoblot analysis, equivalent amounts of protein were loaded in each lane.

### Statistics

All quantitative data are represented as mean ± s.d. p values were generated by Student t-test.

## Supporting Information

Figure S1 FLAG-NGT-FLAG FIT2-V5 constructs are all capable of forming lipid droplets. A, FIT2 constructs are designed as in-frame fusions with carboxyl-terminal V5 and His6 tags. B, HEK293 cells were transfected with each FIT2-V5 construct containing FNF insertions. Mock transfected cells served as a negative control. Cells were stained for neutral lipid and nuclei with BODIPY493/503 and Hoechst 33342, respectively, and analyzed by confocal microscopy, Images are representative of three independent experiments.
(1.14 MB TIF)

Figure S2 Indirect immunofluorescence microscopy for endogenous calnexin topology. A, The known topological model of ER-resident type I integral membrane protein calnexin. B, HEK293 cells were fixed, permeabilized with digitonin with or without Triton X-100, and were incubated with antibodies to detect either the N- or C-terminus of endogenously expressed calnexin. The image is representative of three independent experiments (Bar scale: 5 µm).
(0.75 MB TIF)

Figure S3 FLL(157-9)AAA FIT2 is properly localized to the ER. A. HEK293 cells were transfected with wild-type FIT2-V5 (WT) or FLL(157-9)AAA FIT2-V5 (FLL) and cell lysates processed for Western blot analysis. Expression of WT and FLL were similar. β-actin served as a loading control. B. HEK293 cells were transfected with wild-type FIT2-V5 (WT) or FLL(157-9)AAA FIT2-V5 (FLL). Cells were then processed for immunofluorescence microscopy and stained for FIT2 with an anti-V5 antibody, stained for ER using an anti-C-terminus calnexin antibody and nuclei were stained with Hoechst 33342. Images are representative of two independent experiments (Bar scale: 5 µm).
(1.12 MB TIF)

Figure S4 Specificity of anti-FIT2 LL2 antibody. HEK293 cells were mock transfected or transfected with FIT2-V5, lysed, and analyzed by Western blot using anti-FIT2 LL2. Blot was reprobed with anti-β-actin antibody to control for loading.
(0.70 MB TIF)

Figure S5 Limited trypsin digestion time course of FLL(157-9)AAA and wild-type FIT2-V5. HEK293 cells were transfected with V5-tagged FLL(157-9)AAA (FLL) and wild-type FIT2 (WT), permeabilized with digitonin in the presence of protease inhibitors and proteolyzed for various times with or without trypsin. Proteolysis reactions were quenched with soybean trypsin inhibitor (STI), lysed in its presence, and analyzed by Western blot with anti-FIT2-LL2 antibody. Note the increase in the production of P2 by digestion of FLL-V5 compared to WT.
(0.79 MB TIF)
